# Miniaturized Hybrid Frequency Reader for Contactless Measurement Scenarios Using Resonant Surface Acoustic Wave Sensors

**DOI:** 10.3390/s21072367

**Published:** 2021-03-29

**Authors:** Benedict Scheiner, Florian Probst, Fabian Michler, Robert Weigel, Alexander Koelpin, Fabian Lurz

**Affiliations:** 1 Insititute for Electronics Engineering, Friedrich-Alexander-Universität Erlangen-Nürnberg (FAU), Cauerstr. 9, 91058 Erlangen, Germany; florian.probst@fau.de (F.P.); fabian.michler@fau.de (F.M.); robert.weigel@fau.de (R.W.); 2 Institute of High-Frequency Technology, Hamburg University of Technology, Denickestr. 22, 21073 Hamburg, Germany; alexander.koelpin@tuhh.de (A.K.); fabian.lurz@tuhh.de (F.L.)

**Keywords:** frequency measurement system, SAW sensor, wireless sensors, torque, radio frequency sensors, MEMS sensor

## Abstract

Due to higher automation and predictive maintenance, it becomes more and more important to acquire as many data as possible during industrial processes. However, many scenarios require remote sensing since either moving parts would result in wear and tear of cables or harsh environments prevent a wired connection. In the last few years, resonant surface acoustic wave (SAW) sensors have promised the possibility to be interrogable wirelessly which showed very good results in first studies. Therefore, the sensor’s resonance frequency shifts due to a changed measurand and thus has to be determined. However, up to now frequency reader systems showed several drawbacks like high costs or insufficient accuracy that blocked the way for a widespread usage of this approach in the mass market. Hence, this article presents a miniaturized and low cost six-port based frequency reader for SAW resonators in the 2.45 GHz ISM band that does not require an external calculation unit. It is shown that it can be either used to evaluate the scenario or measure the frequency directly with an amplitude or phase measurement, respectively. The performance of the system, including the hardware and embedded software, is finally shown by wired and contactless torque measurements.

## 1. Introduction

In many areas, it is crucial to measure force and especially torque wirelessly since rotating shafts tremendously increase the wear and tear on connecting devices like slip rings. Therefore, one approach was carried out by battery powered sensor nodes that are used in order to avoid a wired connection [1]. However, this means that the batteries have to be changed after a certain time and an unweight is added because of the additional electronics that is necessary besides the sensor itself. Another way that is shown in [2,3] presents the contactless force measurement with the magnetostrictive principle which is at the moment a very promising approach. Nevertheless, only a small distance between shaft and probe is possible. Additionally, electromagnetic fields, coming for example from electric drives in the surroundings, may interfere with the sensor systems. In the past, the remote sensing approach with LC coupled systems also emerged [4,5,6]. The passive sensor is usually inductively coupled with the reader which then analyzes the impedance variations that are used to determine the actual measurand value. Challenges still exist to make the system more tolerant against a changing distance between the transmitter and receiver coils. Furthermore, surrounding materials can influence the measurement since eddy currents may be induced in nearby metals which may decrease the accuracy. Last but not least, wireless sensor systems that are based on energy harvesting showed good results as well [7,8]. However, since active components are included in the circuits they may be destroyed quickly in harsh environments.

Due to this, different approaches are still required especially for industrial but also e-mobility applications. One of these principles is the usage of resonant surface acoustic wave (SAW) sensors which was already proven to work in the 1990s [9,10]. These sensors show several advantages of being small, contactlessly interrogable, passive and usable in harsh environments. Furthermore, besides the measurement of forces, they can be applied to determine several measurands like pressure, temperature or chemical mixture of liquids or gas, depending on the cut of the crystals of the piezoelectric material that is used as substrate for the sensors and the coating that is applied [10,11,12]. A changing measurand thus influences the resonance frequency of the sensor, which has to be determined, in order to calculate the actual measurand. Several systems in the 433 MHz industrial, medical and scientific (ISM) band showed the feasibility [13,14,15]. However, for commercial industrial applications, this band is not reasonable since it provides a bandwidth of less than 2 MHz and is not approved all over the world. Regarding force measurements, where at least two sensors have to be applied in the same band, this bandwidth is also not sufficient. Furthermore, it should be mentioned that, for the presented demonstrator in [13], two digital signal processors (DSPs) and two RF application specific integrated circuits (ASICs) were used which make the system comparably expensive. With a view to future commercialization, the 2.4 GHz band is a better choice since up to 100 MHz of bandwidth can be used without a license in all countries. Furthermore, another aspect that prevents actual systems from becoming widely integrated in industrial applications are comparably high costs. These originate from high performance analog digital converters (ADCs) and calculation units like field programmable gate arrays (FPGAs) or DSPs, which are necessary to perform the signal processing. Nevertheless, these systems often do not justify the advantages of SAW resonators as sensors [16].

To reduce the costs, six-port based instantaneous frequency measurement (IFM) systems have shown a very promising principle for future frequency readers [16,17]. This approach is used in the presented work and combined with several integrated algorithms and a hybrid concept to create a demonstrator that is more than competitive especially for wireless sensor scenarios. In this article, besides the hardware and software, the results are shown for wired and wireless torque measurements. However, the sensors could be used for other measurands as well.

## 2. Fundamentals

In the following paragraphs, the fundamentals of the two key components are described. Firstly, the sensor type and secondly the principle of the six-port based frequency measurement is explained. Both are necessary to gain a deeper knowledge and to understand the presented system.

### 2.1. Resonant SAW Sensors

**Figure 1:**
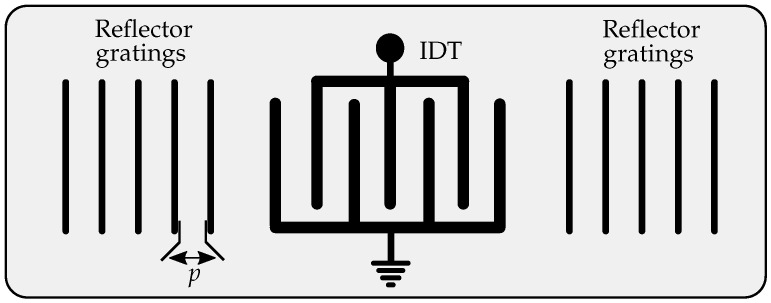
Schematic view of a resonant one-port SAW sensor.

Components in SAW technology are mainly used as band filters in communication systems due to their small size, high quality factor and low insertion loss. Furthermore, these properties can be maintained for devices up to a few gigahertz. It was found out that depending on the cut of the substrate the frequency response can be affected by several environmental parameters such as for example pressure or temperature variations [18]. Due to this, different sensor types based on this technology were developed which can be mainly divided in resonant and delay line SAW sensors [16]. The first type can be described by taking a look at its schematic drawing in Figure 1. During a photolitographic process, different metallic structures are placed on the piezoelectric substrate like quartz (${\mathrm{SiO}}_{2}$) or lithiumniobate (LiNbO${}_{3}$) [11,12,16].

As it can be seen in Figure 1, there are two main structures on the substrate. Firstly, there is the interdigital transducer (IDT), which is in a wireless scenario at one port connected to an antenna, whereas the other one is on ground potential. The IDT is a transducer which transforms an electromagnetic into an acoustic wave and vice versa. Thus, an incoming electromagnetic wave is transformed into a SAW which travels in this case to both sides in the direction of the second structure, the reflector gratings [16]. The specified distance between them, which is denoted as pitch *p*, defines the resonance frequency $f_{0}$ of this resonator [19]. Since the propagation speed of the SAW is much smaller than the speed of light the dimensions can be decreased tremendously. By applying different cuts to the piezoelectric substrate, the dimensions may rather be changed by various measurands like temperature or mechanical stress. Consequently, *p* also changes resulting in a shift of the resonance frequency which thus has to be measured, in order to determine the correct value of the desired measurand. Besides the resonator, the second type is the delay line SAW sensor which uses reflector gratings in various distances. In this case, the delays change as an effect of changing measurands, which is consequently the value that has to be determined. Both types combine many advantages like the possible wireless interrogation, surviving in harsh environments and the passive operating principle.

However, in contrast to the delay line SAW sensors, the resonators show one big advantage which lies in the necessary excitation signal. Whereas the first mentioned sensor type requires a broadband signal for the resonators, a narrowband excitation signal is sufficient, which reduces the complexity of the necessary reader electronic and helps to operate within the specified ISM bands [16]. Consequently, the closer the frequency of the interrogation signal is to the resonance frequency of the sensor, the more power is stored in the resonator and scattered back after a short delay. Nevertheless, especially at the comparably high frequency range of the 2.4 GHz side spurs occur next to the main resonance in the frequency response due to the used substrate materials and manufacturing process. SAW filter designs are not that susceptible to these effects, but, in sensor systems, the measurement accuracy can be affected. However, by applying several features to the presented system, the influence of this effect is significantly reduced.

In particular, the decaying response signal is of high importance for the reader. Generally, it is an exponentially decaying radio frequency (RF) signal with the time constant

(1) $$\begin{array}{c} \tau_{\mathrm{SAW}}=\frac{Q_{\mathrm{SAW}}}{\pi f_{0}} \end{array}$$

which depends on the quality factor of the resonator $Q_{\mathrm{SAW}}$ and resonance frequency $f_{0}$. Consequently, the amplitude of its envelope can be described by

(2) $$\begin{array}{c} A\left(t\right)=A_{\max}e^{-t/\tau_{\mathrm{SAW}}}=A_{\max}e^{\left(-t\pi f_{0}\right)/Q_{\mathrm{SAW}}} \end{array}$$

where *t* denotes the time and $A_{\max}$ the maximum amplitude [16]. The quality factor of the resonant SAW sensors that are used in this work are specified to approximately 2300. With a simplified resonance frequency of $f_{0}=2.45$ GHz, a time slot of only 1 to 1.5 μs can be used for sampling.

### 2.2. The Six-Port Based IFM System

**Figure 2:**
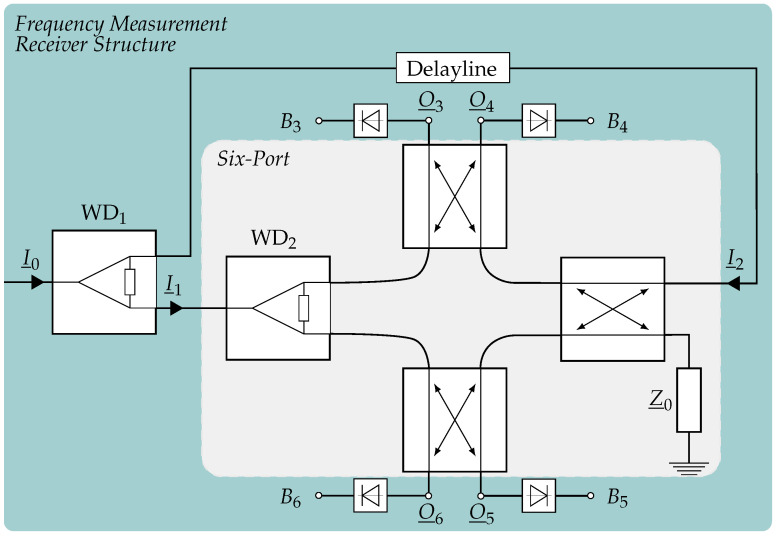
Block diagram of a six-port which is integrated inside a frequency measurement system.

As already mentioned, the six-port receiver is the key structure in this frequency reader which is explained in detail in the following paragraph. The six-port itself as well as the integration into the receiver structure is shown in Figure 2.

In general, it can be described as a differential homodyne I/Q mixer which uses an interferometric principle to generate its four baseband signals. For this system, ${\underline{I}}_{0}$ denotes the incoming complex signal whose frequency has to be determined with the power $P_{0}$, angular frequency $\omega_{0}$ and phase shift $\varphi_{0}$:

(3) $$\begin{array}{c} {\underline{I}}_{0}=\sqrt{P_{0}}e{\;}^{j(\omega_{0}t+\varphi_{0})}. \end{array}$$

By using the power divider WD${}_{1}$, this signal is split up with one part (${\underline{I}}_{1}$) being fed directly into one input port and acting as local oscillator (LO). However, the second part is delayed by a delay line with the delay time $\tau_{\mathrm{dl}}$ which can be calculated from the known length $d_{\mathrm{geo}}$ and propagation speed $c_{\mathrm{dl}}$ as well as from the relative effective permittivity $\varepsilon_{r,\mathrm{eff}}$ and speed of light in free space $c_{0}$, respectively [16]:

(4) $$\begin{array}{c} \tau_{\mathrm{dl}}=\frac{d_{\mathrm{geo}}}{c_{\mathrm{dl}}}=\frac{d_{\mathrm{geo}}\sqrt{\varepsilon_{r,\mathrm{eff}}}}{c_{0}}. \end{array}$$

After the delay, the signal is directed to the other input port and denoted with ${\underline{I}}_{2}$. Due to this and by assuming the delay line and Wilkinson divider WD${}_{1}$ as ideal, ${\underline{I}}_{1}$ and ${\underline{I}}_{2}$ can be written as

(5) $$\begin{array}{cc} \; & {\underline{I}}_{1}=\sqrt{\frac{P_{0}}{2}}\;e{\;}^{j(\omega_{0}t+\varphi_{0})} \end{array}$$

(6) $$\begin{array}{cc} \; & {\underline{I}}_{2}=\sqrt{\frac{P_{0}}{2}}\;e{\;}^{j(\omega_{0}\left(t-\tau_{\mathrm{dl}}\right)+\varphi_{0})}. \end{array}$$

Now, it is obvious that the delay line provokes the frequency dependent phase shift

(7) $$\begin{array}{c} \Delta \Phi =2\pi f\tau_{\mathrm{dl}}=\omega_{0}\tau_{\mathrm{dl}} \end{array}$$

which has to be measured to determine the frequency *f* of ${\underline{I}}_{0}$ by [17]

(8) $$\begin{array}{c} f=\Delta \Phi \frac{c_{\mathrm{dl}}}{2\pi d_{\mathrm{geo}}}=\frac{\Delta \Phi }{2\pi \tau_{\mathrm{dl}}}. \end{array}$$

This can be done by superimposing both input signals on each other with three hybrid couplers and thus four 90${}^{\circ }$ phase shifts which leads to four RF signals ${\underline{O}}_{3}$ to ${\underline{O}}_{6}$ that are differing in power. By using power detectors, the absolute values are generated as the DC-voltages $B_{3}$ to $B_{6}$ which form the differential I/Q signal and thus the complex vector

(9) $$\begin{array}{c} \underline{z}=\left(B_{5}-B_{6}\right)+j\left(B_{3}-B_{4}\right). \end{array}$$

Consequently, the phase shift between the inputs can be calculated with [20]

(10) $$\begin{array}{c} \Delta \Phi =\arg\left(\underline{z}\right)=\mathrm{atan}2\left[\left(B_{3}-B_{4}\right),\;\left(B_{5}-B_{6}\right)\right] \end{array}$$

where the atan2-function is an extension of the usual atan-function, covering now the unambiguous range from −π to π.

Besides this application, the six-port is also used at ISM bands with higher frequencies in radar systems for low-power or high resolution industrial or medical use cases. Here, an unknown displacement is calculated with a known frequency [20,21,22].

## 3. System Concept

**Figure 3:**
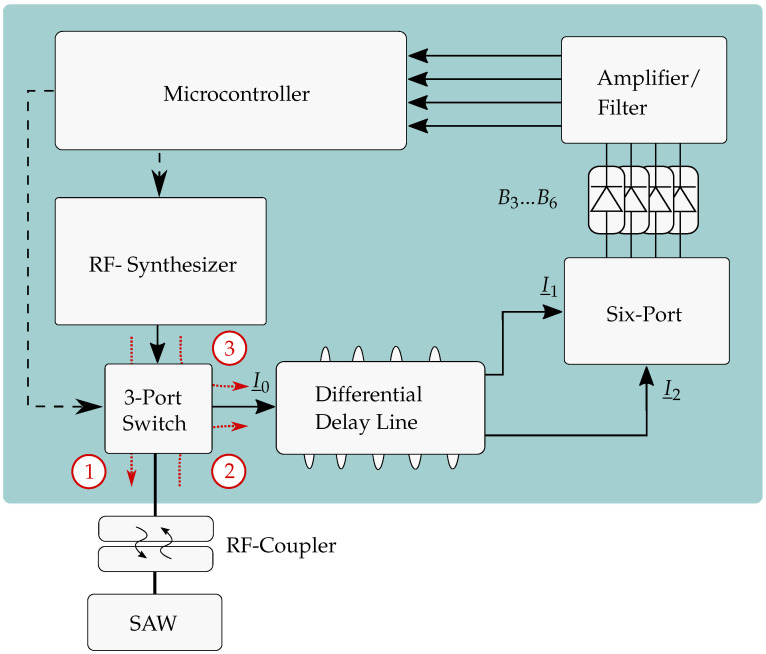
Block diagram of the entire system using a differential delay line. ①: Interrogation; ②: Measurement; ③: Linearization.

After having explained the fundamentals, the basic overview of the system and the integration of the different components is given in this section on the basis of the schematic drawing in Figure 3. It can be seen that the system is not described by a single but a differential delay line which provides several advantages and will be shown in Section 4.3. In the following, the components and their different tasks are explained.

### 3.1. Microcontroller

The microcontroller is responsible for several tasks. First of all, it receives the instructions from the host which is a laptop in the described measurement scenario. Afterwards, these commands are processed and forwarded to the active components that have to be adjusted. Second, the microcontroller includes four dedicated ADCs in order to digitize the differential I/Q signal simultaneously and as a last aspect the frequency is calculated from these values and sent to the host. In [23], a detailed look is taken especially on one main part of the algorithms, the in-situ linearization (ISL), which allows a temperature independent frequency measurement. The basic principle is measuring the phase values of known frequencies and saving them in a look-up table (LUT). The frequency of an unknown signal can then be determined by finding the value in the LUT with the smallest difference to the measured one.

### 3.2. RF Synthesizer

As explained, the RF synthesizer is controlled by the microcontroller and used to generate the signals for the ISL. This means that the frequency and also defined power levels have to be adjusted quickly which is necessary since particularly the detectors’ characteristics are slightly dependent on the input power. As the second task, the excitation signal is generated.

### 3.3. 3-Port Switch

The 3-port switch consists of three highly isolating switches and is accessed by general purpose input output pins (GPIOs) from the microcontroller. Thus, three settings can be applied. Position 1 is used during excitation of the resonant SAW sensor, where in setting 2 the response signal is guided in direction of the receiver. The last switch position 3 is used to directly guide the synthesizer’s signal to the six-port which is consequently the setting for the ISL.

### 3.4. RF-Coupler

Since in this application torque has to be measured wirelessly, an RF coupler around the shaft is required. Therefore, two couplers were presented in the past [24,25] where [25] was characterized regarding its transmission characteristics up to 3300 rounds per minute (rpm). For the measurements in the following that were conducted wirelessly, the coupler from [25] was used in a slightly changed way and will be shown later on page 17.

### 3.5. SAW Sensor

The resonant SAW sensor is connected to the rotary part of the coupler. It receives the interrogation signal, is excited and finally transmits the response signal with the own resonance frequency $f_{0}$ in the range of the ISM band between 2.4 GHz and 2.5 GHz. The sensor should be optimized for high $Q_{\mathrm{SAW}}$ to increase the decay time and for low side spurs to improve the measurement accuracy.

### 3.6. Differential Delay Line

This part of the system replaces the single delay line which was shown for easier comprehension in Figure 2 and is presented for the first time in such a frequency measurement system. In former systems, as presented in [26], an SMA cable of 10 m length was used instead which does not make the system ready for industrial applications. Due to this, now, the delay line was built as well on piezoelectric substrate with the SAW approach because of the much smaller size. However, since delays in the range of 100 ns are difficult to realize and their non-idealities such as cross-talk and triple transit would influence the sensor signal, the differential principle was chosen [27]. This means that the incoming signal ${\underline{I}}_{0}$ is split up into two signals, where both are delayed by a constant delay of $\tau_{\mathrm{Diff}}$ and one by an additional delay of $\tau_{\mathrm{dl}}$. This results in the same phase shift as using a single delay line with the delay time $\tau_{\mathrm{dl}}$. However, due to this, the input signals of the six-port show almost equal powers, which reduces the measurement error. Furthermore, requirements regarding the electromagnetic interference (EMI) between the system components are more easily maintained and the temperature dependency of the delay line as a component is improved.

### 3.7. Six-Port

As a key structure, the six-port represents the receiver structure where ${\underline{I}}_{1}$ and ${\underline{I}}_{2}$ are superimposed. Its evaluation was performed and the structure was used in several other publications before [17,26]. However, the dynamic range particularly depends on the chosen RF detectors. At higher frequencies, the passive components can be designed as planar structures [20,21,22], whereas, at 2.4 GHz, lumped low temperature cofired ceramics (LTCC) components are used which reduce the size significantly.

### 3.8. Amplifier and Filter

After the power detection of the RF signals, four baseband signals are generated. In order to prevent aliasing during the digitization and to improve the level control at the ADCs, the signals are amplified and low-pass filtered, respectively. Afterwards, the signals are guided to the inputs of the ADCs inside the microcontroller where the signals are digitized.

## 4. Prototype

**Figure 4:**
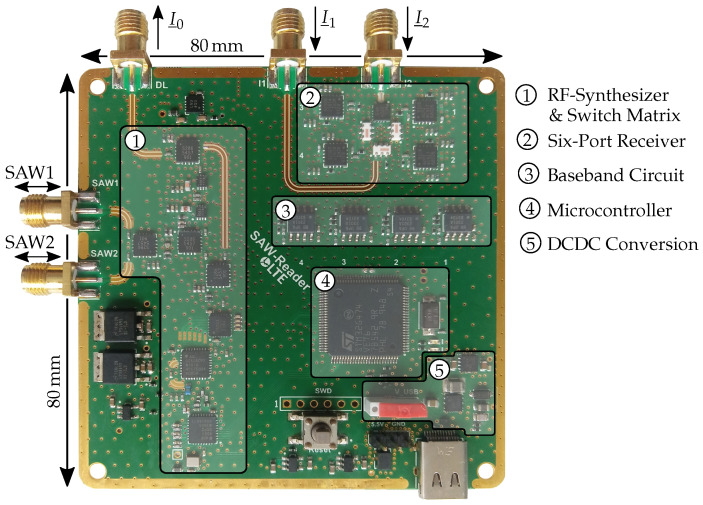
Photo of the realized demonstrator for possible testing of different delay lines.

The section before showed an overview of the system and the tasks of the specific components in theory. Now, in the following part, the realization is explained. In general, the system which is shown in Figure 4 was designed to be compact and low-cost. The printed circuit board (PCB) stack-up was chosen to be four layers with the RF substrate Rogers RO4350B on the outside cores, which shows very good high frequency properties. In between, an FR4 core is applied for stability. The RF transmission lines were laid out in coplanar waveguide with ground plane (CPWG) design to reduce interferences and matched to $Z_{0}=50$ Ω. Additionally, to ensure minimum reflections, the footprints of the SMA connectors were matched to $Z_{0}$ as well. This board was designed to evaluate several types of delay lines which leads to the ports for ${\underline{I}}_{0}$, ${\underline{I}}_{1}$ and ${\underline{I}}_{2}$. It can be furthermore seen that an universal serial bus (USB) interface is implemented for the connection between the board and host. The total costs for all components can be estimated at less than 100 € for a quantity of more than 1000 systems at the time of publication. In comparison to systems with FPGAs and high-performance ADCs, these costs are far below.

In the following, the realization of the different most important parts of the system are described in detail.

### 4.1. Power Management

As a first part, the power management is explained. The circuit is designed in such a way that the system can be powered by either an external source or by most of the USB plugs since a maximum power of 4 W is required. By using a mechanical switch, which source is used can be chosen. During all the measurements, the external source was applied since lower interferences are expected. However, the USB supply showed no apparent differences in comparative measurements.

The maximum voltage of 5 V is necessary for some components. By applying 5.5 V via the external source and using low-dropout regulators (LDOs) with the required output and dropout voltages, the power supply is ensured. It is important to use several LDOs to reduce the EMI between the components and distribute the heat dissipation on the PCB. However, since USB delivers only approximately 5 V, another circuit has to be integrated. In a first step, the input voltage is transformed to 6 V by a DC/DC boost converter, where the layout is crucial in order to keep the output voltage smooth. Afterwards, an LDO is placed which provides the 5.5 V. Both components are chosen to deliver an output current of at least 900 mA. In Figure 4, this circuit can be seen in area 5.

### 4.2. RF-Section and Link Budget

**Figure 5:**
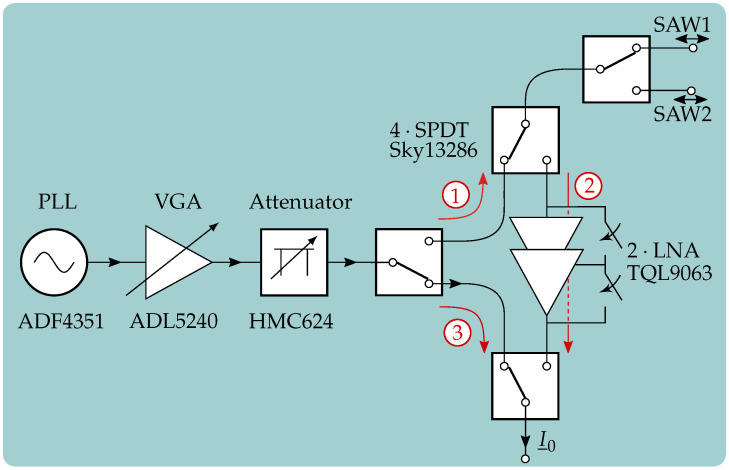
Block diagram of the RF section. Position ①: Interrogation; Position ②: Measurement; Position ③: Linearization.

In this paragraph, the structure of the RF section including signal generation and 3-port switch, which is marked as 1; in Figure 4, is explained. A schematic block diagram can be seen in Figure 5. For the signal generation, a phase locked loop (PLL) is used which is connected to the microcontroller via a serial peripheral interface (SPI). Since a voltage controlled oscillator (VCO) is already integrated in the used ADF4351, the complexity is decreased and only a few components have to be arranged around the integrated circuit (IC). This also includes the loop filter whose bandwidth was set to approximately 120 kHz and is a good trade-off between settling time and phase noise.

For the system, a power amplification as well as attenuation are beneficial in order to improve the signal-to-noise ratio (SNR) and attenuate the signal during the ISL, respectively. However, to get the best possible noise behavior, it is important to stick to the order of components in the signal chain that can be received from the so-called Friis-equation [28]:

(11) $$\begin{array}{c} F=F_{1}+\frac{F_{2}-1}{G_{1}}+\frac{F_{3}-1}{G_{1}G_{2}}+\ldots +\frac{F_{N}-1}{\prod_{n=1}^{N-1}G_{n}}. \end{array}$$

Here, $F_{N}$ and $G_{N}$ denote the noise figure and the gain of the *N*-th element in the signal chain, and *F* represents its overall noise figure. It is obvious that the noise figure of the first stage $F_{1}$ has the biggest influence on *F*. Therefore, the element with the maximum gain and lowest noise figure is chosen in this setup which is the variable gain amplifier (VGA). In this circuit, the component ADL5240 is used, which consists of two components, namely a power amplifier (PA) and an adjustable attenuator, both integrated in the same IC. For the used band, the PA provides a gain of about 19.5 dB and the attenuator a range of 31.5 dB with 0.5 dB step size. It is controllable by six GPIOs that are provided by the microcontroller. However, their order can be changed by external transmission lines. Due to the high gain and equation 11, the amplifier is set as the first component and the attenuator as a second element. Afterwards, the other dedicated attenuator is applied which shows the same specifications as the one that is integrated in the VGA. Consequently, an attenuation range of 63 dB can be used with 1 dB step size if the same six control GPIOs are connected. In the next part, the first single pole double throw (SPDT) switch of the 3-port switch is used to guide the signal either in the direction of the SAWs for excitation (1) or six-port for linearization of the system (3). Besides the three switches for the 3-port switch, another SPDT is used to change between two SAW sensors in order to apply differential measurements. This is, for example, necessary to reduce the temperature dependency a single sensor would have.

When the system is in receiving mode, the SPDT switches are set as it is necessary for the signal to flow as shown with 2. In this receiving path, two cascaded low noise amplifiers (LNAs) are included, where both can be separately added or switched away by an integrated bypass depending on the scenario. Each LNA is specified with a gain of around 16.5 dB in the given ISM band and all SPDT switches as well as the LNAs are controlled by individual GPIO.

**Figure 6:**
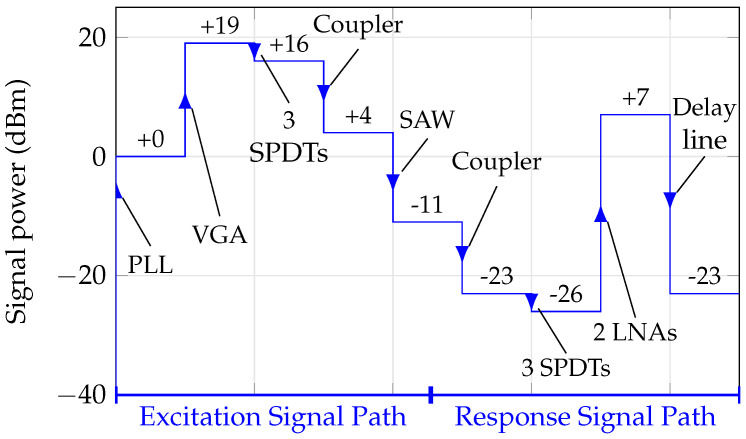
Link budget of the RF section of the proposed frequency reader.

In order to further clarify the gains and attenuations of the different components in the signal path during one excitation and measurement cycle, the link budget is given in Figure 6.

Here, the scenario of using the coupler, which shows an attenuation of up to 12 dB and is presented in [25], is described. Furthermore, it has to be mentioned that the insertion losses and gains are taken from the data sheets. Additional losses due to reflections, transmission lines, and cables are not included in this link budget.

### 4.3. Differential Delay Line

After the RF section, the signal ${\underline{I}}_{0}$ is directed to the delay line. As already mentioned, in this publication, it is the first time that an SAW based differential delay line is integrated in the system. This reduces the size significantly compared to [26] where a single delay line in shape of an SMA cable is used.

**Figure 7:**
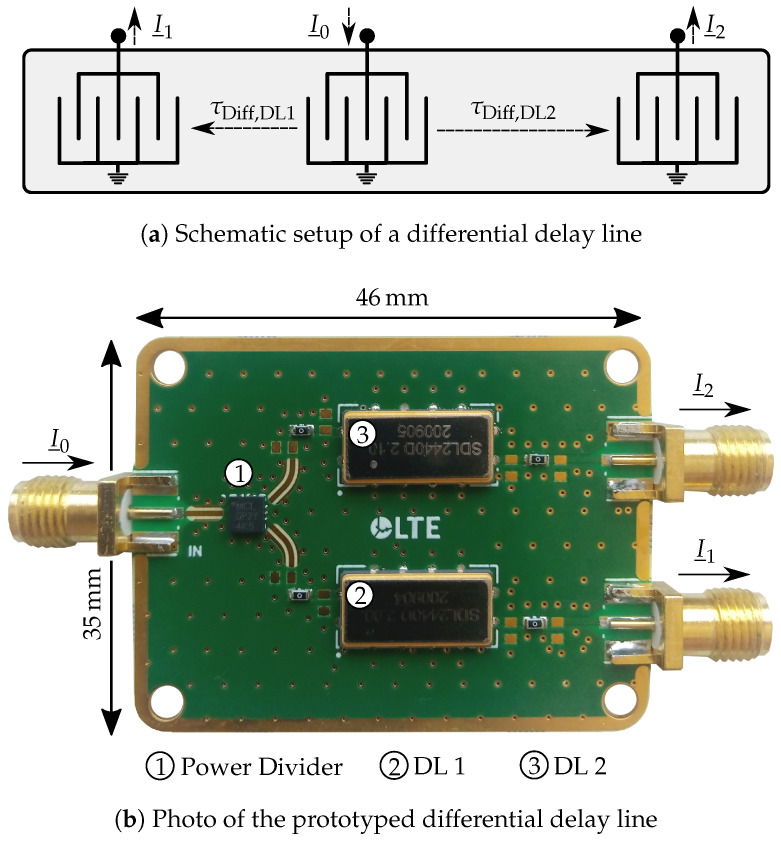
Comparison of the schematic and prototyped version of the differential delay line.

A schematic ideal view and the prototyped version of the delay line are given in Figure 7a,b, respectively. The ideal view in Figure 7a shows how the principle can be implemented on the same die. The incoming signal ${\underline{I}}_{0}$ is directed to the IDT in the middle of the structure where the SAW is excited. The wave then travels to both sides where the other transducers re-transform the acoustic into an electromagnetic wave. Thus, the output signals ${\underline{I}}_{1}$ and ${\underline{I}}_{2}$ are generated that are guided into the six-port inputs afterwards. It can be seen that the delay times at the outputs differ from each other. This means that in this case $\tau_{\mathrm{Diff},\mathrm{DL}1}$ is the time $\tau_{\mathrm{dl}}$ shorter than $\tau_{\mathrm{Diff},\mathrm{DL}2}$:

(12) $$\begin{array}{c} \tau_{\mathrm{dl}}=\tau_{\mathrm{Diff},\mathrm{DL}2}-\tau_{\mathrm{Diff},\mathrm{DL}1}. \end{array}$$

Consequently, $\tau_{\mathrm{dl}}$ corresponds to the delay time of a single delay line in former systems.

In order to verify the operating principle and integration in the system, the realization can be seen in Figure 7b. Due to higher manufacturing cost, this first setup of the differential delay line was done by combining two single delay lines with different delay times and an external power divider. Since the manufacturing is not done on the same die, the component is supposed to have a reduced cross-coupling and only a little higher space requirement. On this realized PCB, the delay times are $\tau_{\mathrm{Diff},\mathrm{DL}1}=2.0\;$μs, $\tau_{\mathrm{Diff},\mathrm{DL}2}=2.1\;$μs and thus $\tau_{\mathrm{dl}}=100$ ns.

**Figure 8:**
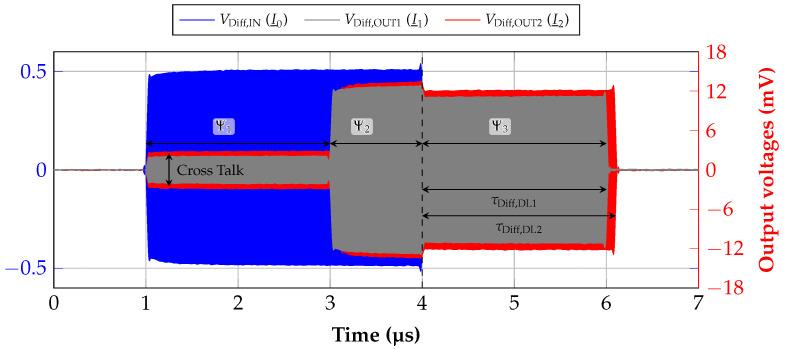
Measured input and output voltages of the differential delay line in the given ISM band caused by an incoming RF pulse.

The characterization of the presented delay line was first conducted outside of the system by a measurement in the time domain. Therefore, the vector signal generator *PSG E8257D* and high-resolution real time oscilloscope *Infiniium UXR0254A*, both from *Keysight* (Santa Rosa, CA, USA), were used. The vector signal generator is connected to an external power divider, where one output serves as reference to the input and one is connected to the input of the delay line. Both outputs of the delay line as well as the reference are fed into the oscilloscope which is capable of easily capturing the signal in time domain in the used ISM band because of its sampling rate of 128 GSa/s and bandwidth of 25 GHz. The results of the measurement, where an RF pulse with a power of 10 dBm and only 3 μs length was applied, are shown in Figure 8. $V_{\mathrm{Diff},\mathrm{IN}}$ represents the reference signal to ${\underline{I}}_{0}$ and both other signals are received from the outputs of the delay line. It can be seen that only cross-talk is received at the outputs in period $\Psi_{1}$ which has the length of $\tau_{\mathrm{Diff},\mathrm{DL}1}$. This effect is based purely on electromagnetic cross coupling. However, after this time, the acoustic wave simultaneously arrives at the output and adds up for the time, the RF pulse is still present at the input. This period is denoted with $\Psi_{2}$. Subsequently, when the pulse is switched off, only the transformed acoustic waves leave the outputs. The period where $\tau_{\mathrm{Diff},\mathrm{DL}1}$ and $\tau_{\mathrm{Diff},\mathrm{DL}2}$ can be measured at the same time is indicated with $\Psi_{3}$. This measurement shows that during linearization the signals have to be provided in a way like this. Finally, the sampling has to be performed in period $\Psi_{3}$ since otherwise the cross talk will reduce the accuracy. Further measurement results with the system in Section 5 will show this behavior more in detail.

### 4.4. Embedded Algorithms

**Figure 9:**
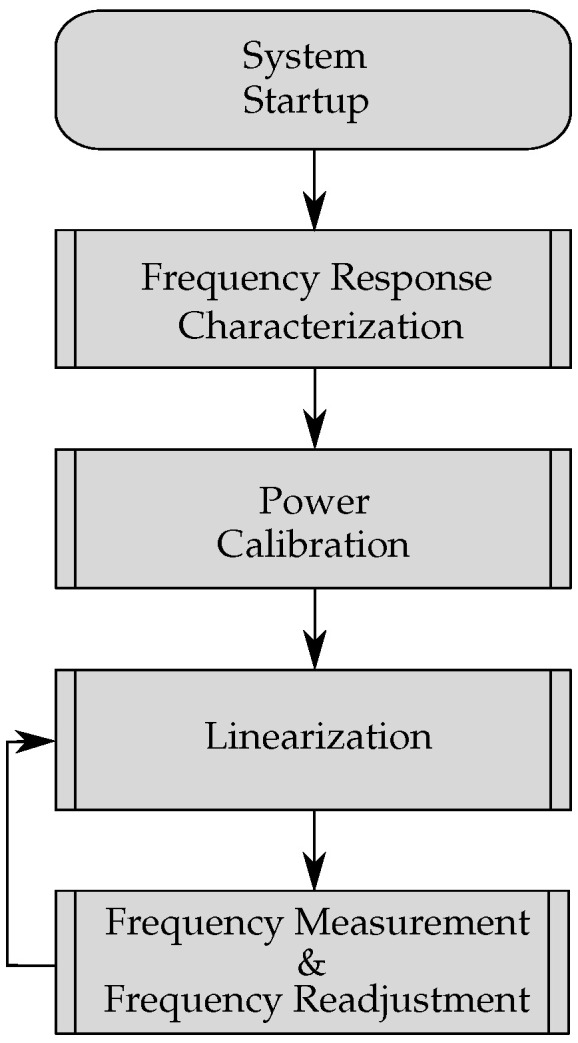
Flow chart of the embedded code after the startup of the system.

Particularly for this system, besides the hardware, the embedded software is also highly important. In the following paragraph, the different algorithms are described with the help of the flow chart in Figure 9.

After the system is turned on and received the command from the host where to expect the frequency $f_{0}$, the microcontroller starts with the first main algorithm which is called frequency response characterization. The basic principle was firstly presented in [29], however, with the calculation examined on an external device. This algorithm serves to coarsely find the main resonance frequency of the sensor in an unloaded situation. This is done by a power measurement of the response signal which means that it interrogates the sensor in the defined frequency band with a given frequency step size. At each frequency, the power of the response signal is measured by adding up the values of the four output detectors which thus gives a relative power characteristic over the frequency range. The frequency with the maximum measured power can be regarded as $f_{0}$. Since the frequency response of an SAW sensor may shift due to several environmental changes while the system is off, this algorithm is highly important.

In order to perform the ISL correctly, the phase values which are stored in the LUT have to be measured at a similar power level as the response signal of the SAW. Due to this, the power calibration can be performed to reduce the power steps inside the LUT during the ISL. This results in lower calculation time and less memory usage which is especially important on low-cost embedded devices with limited memory. Furthermore, the power settings for the ISL are chosen by the system itself depending on the scenario—for example, if a coupler with higher insertion loss or if short cables are applied. This means that the attenuators are set to values for the signals during the ISL to get similar powers as which are received from the actual response signal of the SAW sensor. The process is described as follows. At first, the resonator is excited at $f_{0}$ which was determined by the frequency response characterization and afterwards the power is measured and stored. Second, the 3-port switch is switched to 3 and the values of the attenuators are changed as long as the measured power in the six-port matches the power of the response signal. If a scenario of varying attenuation is expected, for example due to the change of the distance in free space, further power steps can be chosen around the calibrated power value.

After determining the two parameters, namely the resonance frequency and the relative power the system starts with the first full linearization process which was presented in [23]. The specified resonance is now taken as the center of a frequency range which is about 1.5 times larger than the actual possible frequency shift in order to make sure that the sensor does not leave the linearized band. The relative power which was determined in the step before is now taken to be the power level for this linearization. Consequently, the frequency range is linearized with the predefined frequency steps and the phase values are stored in an embedded LUT.

Following the completion of this table, one can move on to the next step, which is the frequency measurement and afterwards also the readjustment of the interrogation frequency. For the first measurements, the sensor is excited at the determined $f_{0}$ and the phase of the response signal is calculated from the resulting four baseband signals. Additionally, the matching value is found in the LUT and then sent to the host. However, it has to be kept in mind that a changing measurand causes the entire frequency response to shift in the same direction. As a consequence, the main resonance is charged with less power since it moves away from the interrogation frequency. This means that the SNR decreases as the response signal is also of less power. Moreover, by sticking to this single interrogation frequency, the influence of the side spurs increases which leads to a nonlinear function or even a totally wrong measured frequency. Both problems can be reduced by readjusting the interrogation frequency if the measured frequency changes too much. As shown in [30], where this algorithm was presented for the first time as a single building block, it keeps the SNR on a constant level and tremendously reduces the influence of side spurs. In order to make the system more tolerant against temperature changes, the phase of a single frequency value can be re-linearized after measuring one frequency point.

## 5. Measurements

**Figure 10:**
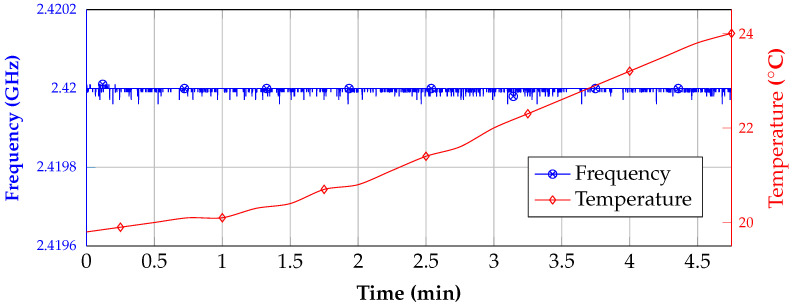
Frequency measurement during a changing temperature and with a re-linearization of the system at the same time.

This re-linearization process was proofed with the results of the first measurement, which are shown in Figure 10. For the setup, the system was placed in a temperature chamber and the frequency of the internal synthesizer was measured continuously while the temperature was increased. Furthermore, the temperature was recorded with a sensor that was placed on the differential delay line. It can be seen that, even though the temperature rises, the frequency only changes in a narrow band of 40 kHz. This is a high improvement in comparison to the results that are presented in [23], where only a single linearization was conducted previous to the measurement, since with each frequency measurement a single value in the LUT is re-linearized the duration until the LUT is up to date highly depends on the measurement update rate. Thus, by increasing the update rate, the temperature dependency will also further improve.

In the following section, further measurements are presented that were conducted with the proposed system. If not mentioned differently, an averaging of 128 measurement values was used, which leads to an update rate of 14 Hz.

**Figure 11:**
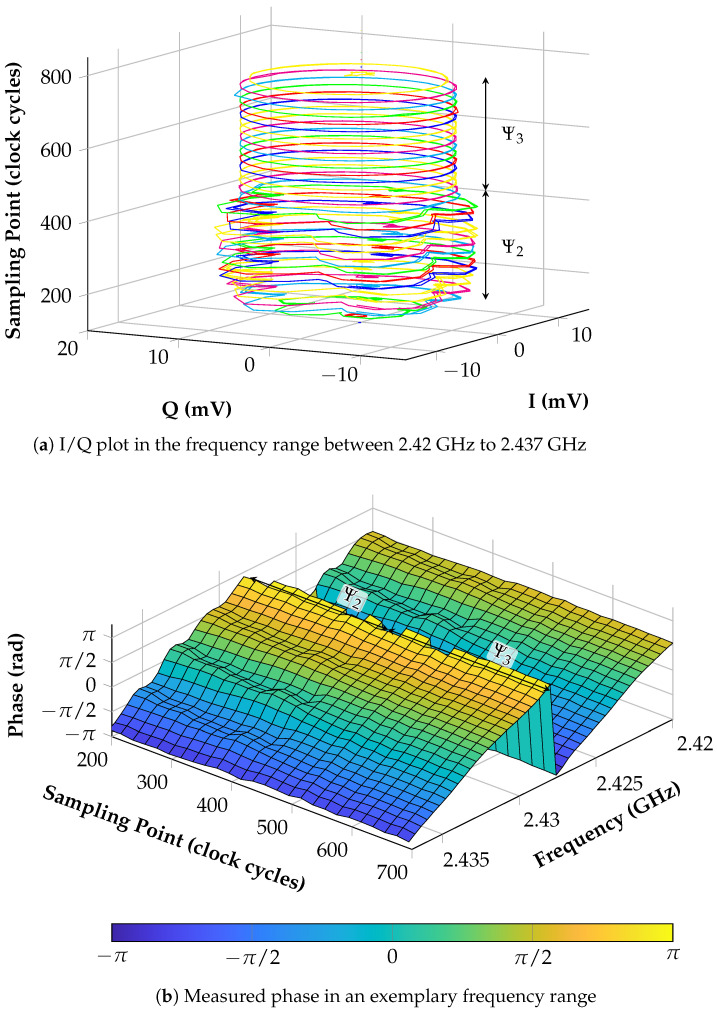
I/Q plot and phase measurement during the linearization process depending on the point of sampling. One clock cycle lasts 5.88 ns.

With a first measurement, the importance of the sampling point during the linearization is illustrated if the differential delay line is used. Consequently, this means that for this measurement no SAW sensor is needed. This is shown and explained in this article with Figure 8 where it could be clearly seen that only $\Psi_{3}$ is the period where the signal is free of cross-talk and sampling should be done. To demonstrate the systems’ performance, this is visualized with Figure 11.

In Figure 11a, the raw baseband signals during a linearization process are added together in an I/Q plot. In this case, the frequency range from 2.42 to 2.437 GHz is shown due to illustration reasons, and it can be seen that the sampling point of the microcontroller’s ADCs is shifted. This point is defined by a hardware trigger that derives from an integrated timer. By using this sampling method, it is possible to guarantee that all four ADCs sample simultaneously and that the sampling point can be defined with a resolution of one clock cycle. In contrast to the usage of interrupts, the hardware trigger is much faster and provides no overhead which could block other processes. During the performed measurement, one clock cycle is 5.88 ns long since a clock frequency of 170 MHz is used. The periods $\Psi_{2}$ and $\Psi_{3}$ that were described with Figure 8 are also denoted here. It is obvious that, during period $\Psi_{2}$, the circles in the I/Q plot show several edges and nonlinearities which come from the superposition with the static cross-talk. However, this changes immediately by reaching period $\Psi_{3}$, meaning that the cross-talk disappears since no RF signal is applied at the input of the delay line.

The influence of this electromagnetic interference and cross coupling can be further clarified by taking a look at Figure 11b where the calculated phase is presented since this is the value which is stored in the LUT. Again, the measurement was conducted for a range of different clock cycles and frequency values. In the actual application, the LUT would only include the values for one sampling point which consequently shows how an incorrectly chosen timer period for triggering the ADCs can degrade the measurement result. Thus, it is obvious that many nonlinearities can be seen in shape of ripples during $\Psi_{2}$, which are not present during $\Psi_{3}$. This again proves that only the last period can be chosen for sampling during the linearization. Moreover, it can be said that a further specification of the sampling point is not needed.

**Figure 12:**
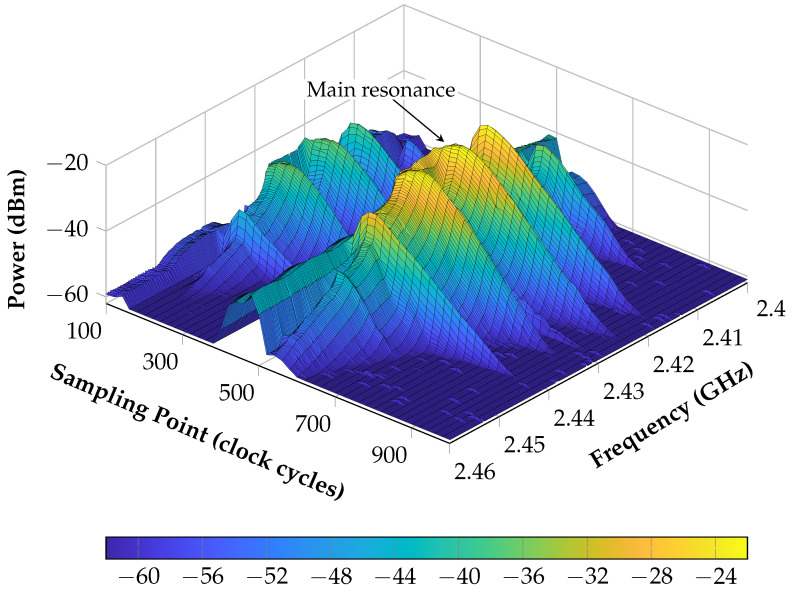
Characterization of the resonant SAW sensor by measuring the power at several frequency points and for equally spaced range of sampling points. One clock cycle lasts 5.88 ns.

In an additional measurement, the hybrid concept is now explained and the high performance of the system is verified. This is done by evaluating the amplitude of the decay of an SAW sensor with the proposed system and differential delay line over a certain frequency and clock cycle range. Therefore, a sensor is connected by a cable to one SAW port of the system. Afterwards, the already explained algorithm for frequency response characterization is performed again for an equally spaced array of clock cycles and the values are averaged over 64 measurements. The measurement results can be seen in Figure 12.

As a first aspect, it is obvious that two decays can be seen directly after each other where the first shows about 15 dB less power than the second. This difference comes from the cross-talk over the delay line and attenuation which can be specified to 45 dB and 30 dB, respectively. Again, this illustrates that, during the measurement, it is absolutely necessary to know exactly when to sample. Here, this is even more important than during the linearization procedure, since a shifted sampling results directly in a changed signal power and thus decreases the SNR.

Secondly, it can be clearly seen that the resonator does not show an ideal behavior with only one resonance. In total contrast to this, multiple side spurs can be detected next to the main resonance at 2.42 GHz which shows slightly the longest decay that thus corresponds to the highest quality factor.

As a result, by using this characterization the entire system can be specified in multiple domains, which are the SAW sensors, the delay line and the interface between the sensor and the reader. Due to this, different statements can be achieved. One aspect is that the characterization of new SAW sensors, for example with less side spurs, is strongly simplified where usually expensive measurement equipment is necessary, meaning that the frequency behavior is characterized for several sampling points. Moreover, different types of delay lines can be compared to each other regarding the attenuation and delay times. All of these aspects can be evaluated in the same system that is used for the frequency measurement as well and thus provides a huge advantage. Furthermore, this can also be used in the future to let the system characterize the sensor, delay line, and attenuation of the coupler on its own if further algorithms are applied.

As a final measurement, the frequency of an SAW sensor is determined during a torque measurement scenario since in contrast to other parameters like temperature or chemical mixture of liquids it can be quickly and accurately applied and referenced. In a first step, the measurement setup is explained and results for a wired and finally for a contactless scenario are presented.

**Figure 13:**
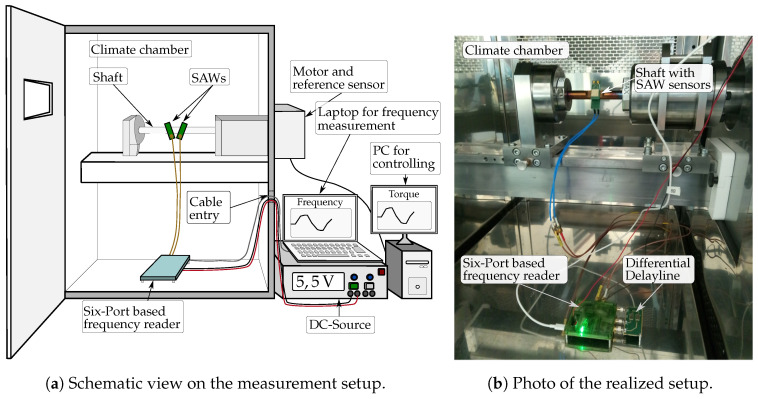
Schematic and realized presentation of the measurement setup for torque measurements inside a climate chamber.

The measurement scenario is shown in Figure 13 in two ways where the focus is first on Figure 13a in which the schematic presentation of the setup is given. The system is placed inside a climate chamber with constant temperature in order to focus only on the measurement performance of the system. The chamber is equipped with a shaft and attached SAW sensors for a differential measurement. It can be seen that the shaft is locked on the left side and connected to the motor and reference sensor, a strain gauge, which are both placed outside of the chamber. By using a PC, different torque profile patterns can be applied and are recorded with the data from the reference sensor. The reader is further connected to the SAW sensors via SMA cables. The power supply is ensured by an external DC source which provides a voltage of 5.5 V, and the data connection to a laptop is realized via USB where all cables are guided through a cable entry. Moreover, the system is controlled via Matlab on the laptop which shows the received frequency values in a live graphical user interface.

In Figure 13b, the realized setup in the climate chamber Vötsch ${\mathrm{VC}}^{3}$ 4034 is depicted. The delay line that is matched with its outputs to the frequency reader can be seen as well.

**Figure 14:**
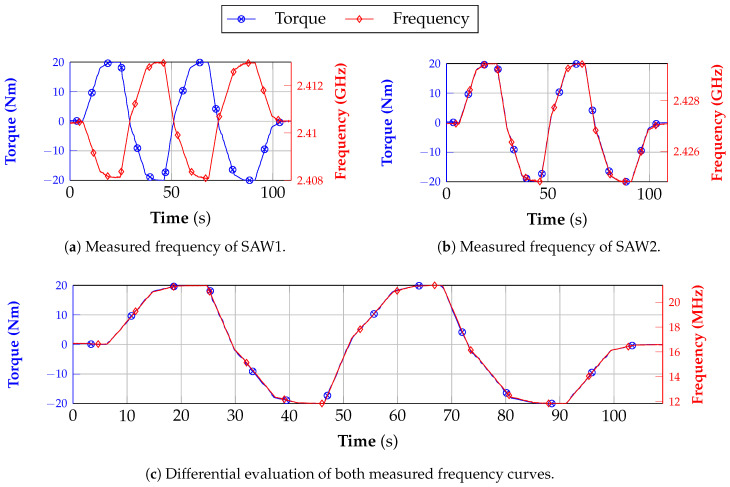
Comparison between the measured torque and frequency which were obtained by the reference sensor and frequency reader, respectively.

The corresponding measurement results for this particular setup are shown in Figure 14. First, a torque sequence is conducted where starting from unloaded condition 20 Nm and −20 Nm are applied two times after each other and finally the initial status is reached again. This sequence was performed in a dynamic way and takes almost 2 min. At the same time, the frequency is measured and saved on the laptop. In a post-processing step, the two curves of the SAW reader and reference are synchronized due to the different PCs.

In Figure 13a,b, the frequency profiles of the individual SAW sensors are depicted. The differential behavior is obvious since both curves proceed in an opposite way. While the resonance frequency of SAW1 decreases with rising torque, $f_{0}$ of SAW2 increases. The subtraction of both signals finally results in the curve that is presented in Figure 14c. It can be seen that the frequency curve matches extremely well with the recorded torque from the reference. Furthermore, no difference between the rising and falling edge of the frequency profile can be detected which could otherwise indicate a mechanical hysteresis in the structure.

**Figure 15:**
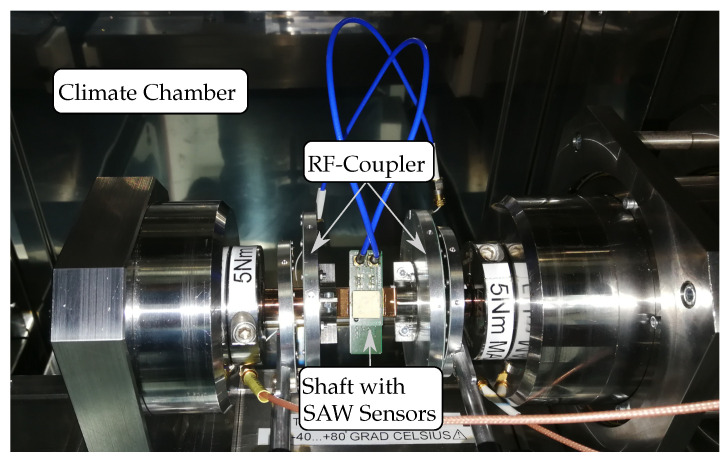
Photo of the wireless measurement setup using an RF coupler.

In order to evaluate the characteristics during a contactless scenario as well, another measurement was performed. Therefore, a closer look is taken to the changed setup of the shaft in Figure 15. The SAW sensors are again attached to the shaft but in contrast to the measurement before each sensor is now connected to one rotating part of the coupler that was presented in [25]. On the opposite side, the static part is connected to the reader port.

This enables a contactless scenario where even high rotating speeds of up to 3300 rpm would be possible with an appropriate measurement setup. It has to be mentioned that in this figure the coupler disks are shown with more space in between due to better visualization. During the measurement, the distance was determined to be between 2.5 and 3 mm. Even though the coupler was specified with 12 dB maximum insertion loss, small changes in the design, the added cables and distance increased it to be 18 dB for each path. This means that, compared to the wired scenario, the SNR decreases by about 2×18dB−16dB=20 dB since the second LNA is used in this case.

**Figure 16:**
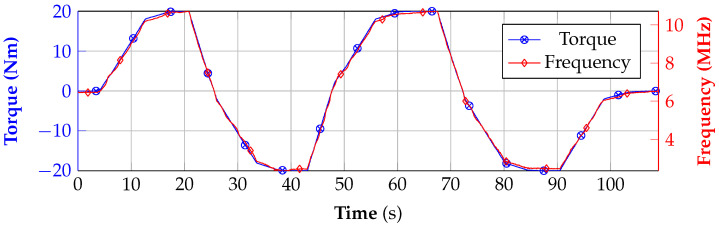
Measurement results of the contactless scenario and comparison to the reference.

The results of the differential measurement, which are given in Figure 16, show that, in spite of the loss of signal strength, the signal is still in very good agreement with the reference data. However, small variations can be seen compared to Figure 14c. This problem can be resolved by applying another PA in the transmit-path to increase the signal strength depending on which path loss is expected with the used application.

**Figure 17:**
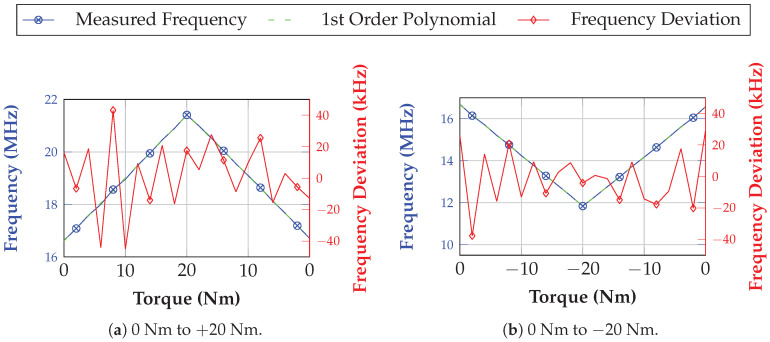
Measurement results for a semi-static measurement scenario for negative and positive torque values. The step width is 2 Nm where at each point an averaging of 20 measurement values was conducted. The frequency deviation from a fitted first order polynomial is shown.

In order to take a closer look to the accuracy of the system, a further measurement was performed during a wired non-dynamic scenario. This means that the torque range between −20 Nm and 20 Nm was covered by equally spaced measurement points with a step size of 2 Nm. At each step, 20 measurements were performed. During the post-processing, these values were averaged and a first order polynomial was fitted through as it is depicted for positive and negative torque values in Figure 17a,b, respectively. It can be clearly seen that maximum deviations of ±40 kHz exist. However, this is still depending on the accuracy of the reference sensor and can also be improved by better SAW technology with an improved side spur behavior.

## 6. Discussion

In this article, a frequency reader demonstrator for resonant SAW sensors was presented with a frequency calculation that is entirely embedded on a single microcontroller. In addition, the integration of a differential SAW based delay line and the hybrid concept for evaluating the entire system including the SAW sensor were presented for the first time. Previously presented algorithms were merged in this system, which leads to way more robust results regarding the side spurs. It was shown that the system’s measured frequency profile is in very good agreement with the applied torque and that, due to its low-cost approach, this principle is applicable for different high-volume sensor markets. Additionally, the re-linearization of the system during the measurement showed the possibility to make the system less susceptible to variations of external parameters. By further optimization of algorithms like the ISL, the sampling rate can be significantly improved in the future. Moreover, the system can be even more decreased in size by integrating several parts like the RF section or six-port in ASICs [31] which consequently results in ultra-compact and low-cost sensor systems. Use cases can be, for example, the wireless determination of measurands as temperature, chemical compositions of liquids or gases, pressure, strain or torque in applications in industry, e-mobility or other harsh environments.

## Data Availability

Not applicable.
